# Effect of Multisession Progressive Gait-Slip Training on Fall-Resisting Skills of People with Chronic Stroke: Examining Motor Adaptation in Reactive Stability

**DOI:** 10.3390/brainsci11070894

**Published:** 2021-07-07

**Authors:** Shamali Dusane, Tanvi Bhatt

**Affiliations:** 1 Department of Physical Therapy, College of Applied Health Sciences, University of Illinois, Chicago, IL 60612, USA; sdusan2@uic.edu; 2 Ph.D. Program in Rehabilitation Sciences, Department of Physical Therapy, College of Applied Health Sciences, University of Illinois, Chicago, IL 60612, USA

**Keywords:** multisession training, stroke, adaptation, fall-risk, gait-slip

## Abstract

Background: This study examined whether a multisession gait-slip training could enhance reactive balance control and fall-resisting skills of people with chronic stroke (PwCS). Methods: A total of 11 PwCS underwent a four-week treadmill-based gait-slip training (four sessions). Pre- and post-training assessment was performed on six intensities of gait-slips (levels 1–6). Training consisted of 10 blocks of each progressively increasing intensity (four trials per block) until participants fell at >2 trials per block (fall threshold). In the next session, training began at a sub-fall threshold and progressed further. Fall outcome and threshold, number of compensatory steps, multiple stepping threshold, progression to higher intensities, pre- and post-slip center of mass (CoM), state stability, clinical measures, and treadmill walking speed were analyzed. Results: Post-training, PwCS demonstrated a reduction in falls and compensatory steps on levels 5 and 6 (*p* < 0.05) compared to pre-training. While an increase in pre-slip stability was limited to level 6 (*p* < 0.05), improvement in post-slip stability at lift-off was noted on levels 2, 3, and 5 (*p* < 0.05) along with improved post-slip minimum stability on levels 5 and 6 (*p* < 0.05). Post-training demonstrated improved fall (*p* < 0.05) and multiple stepping thresholds (*p* = 0.05). While most participants could progress to level 4 between the first and last training sessions, more participants progressed to level 6 (*p* < 0.05). Participants’ treadmill walking speed increased (*p* < 0.05); however, clinical measures remained unchanged (*p* > 0.05). Conclusions: Multisession, progressively increasing intensity of treadmill-based gait-slip training appears to induce significant adaptive improvement in falls, compensatory stepping, and postural stability among PwCS.

## 1. Introduction

About 800,000 people in the United States suffer from stroke each year [1]. Stroke as a critical cardiovascular accident has been associated with long-term disability and mortality [1,2]. Falls are a common complication among people with chronic stroke (PwCS) on exposure to external environmental perturbations, especially during walking [3,4,5]. The reported fall incidence rates during the chronic phase post-stroke ranges from 23% to 50% [6], with the majority of fallers suffering from varying severity of fall-related injuries [7]. PwCS are known to be twice at risk of falling compared to age-matched healthy older adults [8]. Such greater fall-risk has been associated with stroke-induced balance deficits and gait impairments [3,9,10]. The balance control system comprises of proactive control, which maintains or restores postural equilibrium from self-induced perturbations, and reactive control, which is associated with the ability to restore a state of postural equilibrium following external perturbations [11]. Reactive balance control via a compensatory stepping response or a change-in-support reaction has been well-established as an essential strategy for avoiding falls following sudden, unexpected externally-induced balance loss [12,13]. Consequently, there is a need to develop an effective fall-prevention intervention that specifically focuses on improving impaired reactive responses in PwCS [14,15,16].

Systematic reviews performed on neurologically intact healthy adults have indicated the emergence of perturbation training as a task-specific balance training paradigm involving exposure to repeated perturbations, thereby simulating real-life loss of balance [17,18,19]. Previous evidence has shown promising improvements in reactive balance control, postural stability, and compensatory stepping strategies resulting in fall-risk reduction among healthy older adults [20,21,22,23,24]. Along similar lines, preliminary perturbation training studies have been performed on PwCS, employing mid- to large-magnitude treadmill-based perturbations [25,26,27]. These single-session training studies have demonstrated the preserved ability of PwCS to undergo reactive adaptation by following a fixed protocol with a standardized perturbation training dosage (intensity and number of trials) for all participants—irrespective of the severity of their stroke-induced sensorimotor impairment—thus leading to reduced fall-risk.

Recently, several multisession perturbation training paradigms involving an exposure to more training sessions with a greater number of perturbations have been used to improve recovery responses among individuals post-stroke [28,29,30,31,32,33]. These studies have demonstrated a significant improvement in the percentage of successful recovery [29], multiple stepping threshold [30,33], leg angles [31], steady-state gait characteristics [28], and predicted fall-risk [28], along with a decreasing trend in daily falls [32]. Additionally, a significant improvement in clinical balance measures such as the Berg Balance Scale, the Activities-Specific Balance Confidence Scale, and a reactive component of the Mini Balance Evaluation Systems Test (Mini-BESTest) were also found [30,31,32]. Thus, these multisession perturbation training studies demonstrated promising results in laboratory outcomes and clinical measures.

However, these multisession perturbation training studies have been associated with certain limitations. About four out of the six multisession studies employed stance perturbations, while only two multisession studies in PwCS involved gait-perturbation training [28,30]. In view of the dynamic nature of walking, gait-perturbations impose different task demands on both non-paretic and paretic limbs and are more challenging than stance perturbations. Given the relatively few studies examining gait-perturbations in PwCS [34,35,36] there is a need to establish strong evidence on the effects of gait-perturbation training. Secondly, these multisession studies did not quantify the training dosage based on participants’ impairment. A study by Bhatt et al. [25] examined the effect of motor impairment on slip-like stance perturbation training in PwCS and suggested that providing perturbation training at an intensity appropriate to the level of sensorimotor impairment is important for inducing adaptation effects. Besides, with a higher incidence of falls during walking among PwCS, providing task-specific training with gait perturbation might be ecologically more valid to reduce real-life falls as compared to stance perturbations.

Moreover, these studies provided training using low magnitude perturbations such that Mansfield et al. [32] and Schinkel-Ivy et al. [33] provided manual push–pull perturbations, while Handelzalts et al. [30] and Punt [28] used low magnitude accelerations. Furthermore, greater adaptive gains with high-intensity training have been suggested from past locomotor studies in the neurological population [37,38,39,40], and gait-perturbation studies in healthy populations [41,42,43]. Thus, considering the large magnitude of real-life external environmental perturbations and the crucial role of training intensity in adaptation, it can be claimed that the training intensity employed by these studies was not quite challenging. Lastly, all of the multisession studies failed to determine the underlying mechanism of induced adaptation and did not account for any pre–post changes in mechanistic factors such as proactive and reactive postural stability, which have been established as crucial biomechanical contributors of fall prevention [16].

However, PwCS with varying degrees of impairment may not safely tolerate high-intensity gait-perturbation training. Progressively increasing the intensity of training might be more feasible and a second-best alternative to inducing adaptive changes after higher intensity training, as demonstrated by studies in older adults [25,41]. Initiating training with a gradual increase in perturbation might be better suited to accommodate participants’ sensorimotor and reactive balance impairments. Such an approach of progressive overload, based on the principles of motor learning, might induce greater gains and more robust reactive adaptation in PwCS [44]. This pre–post design study aimed to determine the effects of a four-week multisession, progressive treadmill gait-slip perturbation training protocol on the fall-resisting skills of PwCS. This study also examined the changes in clinical outcome measures and the walking speed post-training among PwCS.

## 2. Methods

### 2.1. Participants

**Figure 1:**
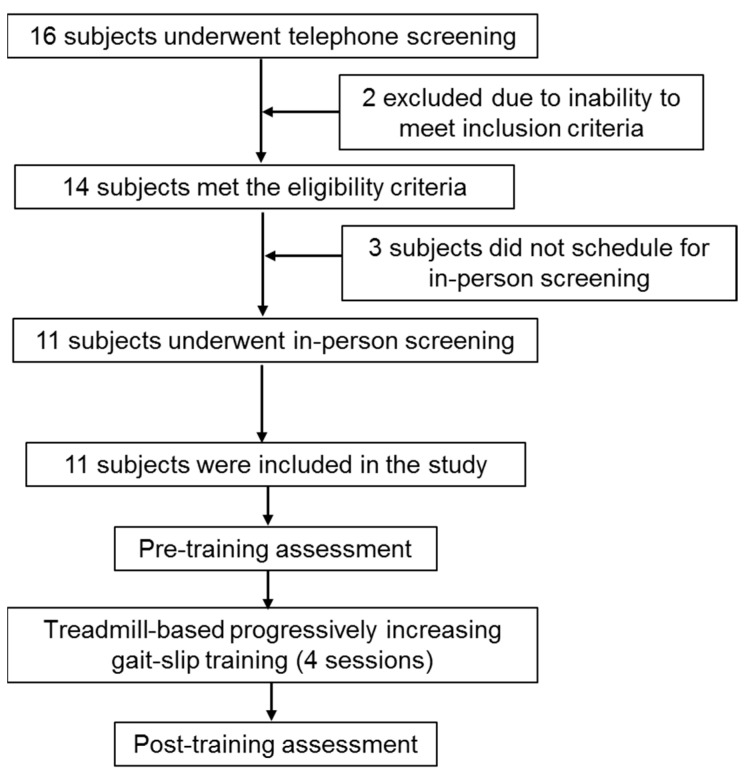
CONSORT flowchart summarizing participant recruitment, screening, and training for the data presented in this manuscript.

A total of 11 community-dwelling PwCS more than six months post cortical stroke (as confirmed by a physician)—with an ability to ambulate independently, with or without an assistive device—were included in the study. Participants with a cognitive score of ≤26/30 on the Montreal Cognitive Assessment Scale, an aphasia score of ≥71/100 on the Mississippi Aphasia Screening Test, bone density of <−2 (T score) on the ultrasound, or the presence of any self-reported neurological, musculoskeletal, or cardiovascular conditions during the in-person screening were excluded. Chronicity of stroke, severity of motor impairment using the Chedoke–McMaster Stroke Assessment scale, balance measures using the Berg Balance Scale (BBS), Mini Balance Evaluation Systems Test (Mini-BESTest), activity confidence on Activities-Specific Balance Confidence Scale (ABC), gait-speed using the 10-m walk test, and cardiovascular endurance testing using the 6 min walk test were assessed. All assessments were also repeated post-training. The demographic details of the participants are presented in Table 1. Before enrolment in the study, all participants consented and were asked to sign the consent form as approved by the institutional review board of the University of Illinois at Chicago (UIC). This study was performed at Cognitive Motor Balance Rehabilitation Laboratory at UIC from January 2020 to September 2020. Figure 1 shows the CONSORT flow diagram for this study.

### 2.2. Training Protocol

**Figure 2:**
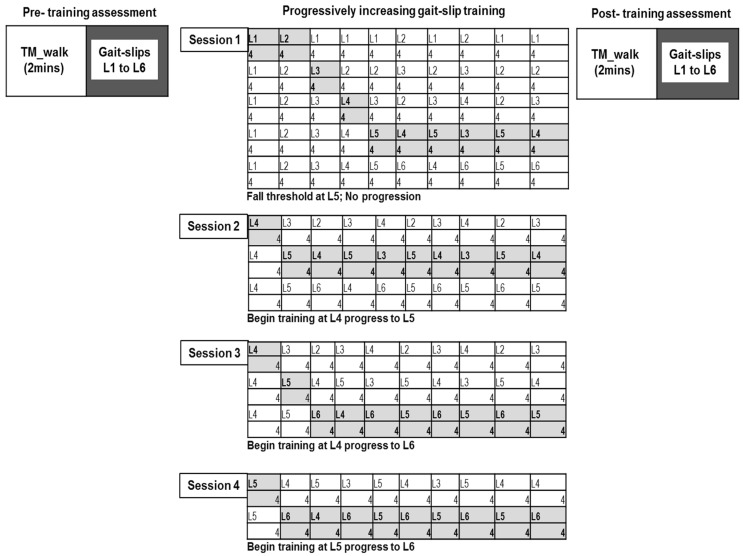
Testing and training protocol for the multisession treadmill gait-slip paradigm. Participants underwent progressively increasing treadmill gait-slip training that consisted of 10 training blocks (40 perturbations); training intensity was increased when the participant was able to successfully recover and experience less than two falls in each block of four trials. Progression was continued until fall threshold was reached. Pre-training and post-training assessment included exposure to all intensities (L1 to L6) of treadmill-based gait-slips at the participants’ self-selected walking speed.

All participants underwent four-weeks of treadmill-based progressively increasing gait-slip training, performed once per week using the ActiveStep (Simbex) motorized treadmill (four sessions). Pre- and post-training assessment was performed on six different intensities of gait-slip to determine the training effect (level 1 to 6) (Figure 2). A safety harness was firmly attached to the participants to prevent them from touching the treadmill belt in the event of a fall during training and assessment. Participants were instructed to try to recover from loss of balance and to avoid falling.

### 2.3. Assessment

Participants were asked to walk for two minutes on the moving treadmill belt at their natural walking speed in order to become familiar with treadmill walking; the comfortable walking speed was then recorded. After this, the participants were subjected to all six intensities of walking slips, from lower to higher intensity (L1 to L6) at their self-selected walking speed (determined previously from the 10 m walk test) (Figure 2).

### 2.4. Intervention

Our paradigm provided task-specific gait-slip training to reduce fall-risk during walking, as this is the activity when most falls occur among PwCS [3,4,5]. Providing greater training dosage to induce adaptive changes might be beneficial in PwCS, given their slower rate of locomotor adaptation compared to neurologically healthy controls [45]. Previous studies in healthy older adults are suggestive of training with more trials (40 vs. 24) to enhance motor learning and retention [46]. Similarly, our training consisted of 40 slips per session, which aimed to intentionally overtrain a task (overlearning) for better retention effects [47,48]. While dose-response studies for perturbation training among healthy adults have shown optimal results using high-intensity perturbations (more repetitions and large magnitude) and low frequency (fewer training or booster sessions) compared to low-intensity and high-frequency training [41], such training may not be well tolerated by PwCS. Accordingly, our training paradigm provided progressively increasing intensity of perturbation training, which was the second best alternative to high-intensity training in an attempt to make our paradigm superior [41]. Thus, our paradigm was designed based on the principles of motor learning.

The training included a total of four training sessions on an ActiveStep (Simbex) motorized treadmill, one session per week for four weeks. The training consisted of 10 blocks for each perturbation intensity, with four trials in each block, resulting in a total of 40 gait-slips (Figure 2). Training began with the lowest intensity and progressively increased until the participant experienced more than two falls on the given block, which was termed as the fall threshold. After reaching the fall threshold, training was not progressed any further, and participants were trained at an intensity lower than the fall threshold. In the next session, training began with an intensity lower than the fall threshold, and if less than two falls occurred, the training intensity was progressed. Treadmill gait-slip perturbations consisted of an acceleration of 3 m/s^2^ for gait-slip intensity level 1 to level 6. The slip distance for levels 1, 2, 3, 4, 5, and 6 were 1.5 cm, 3.37 cm, 6 cm, 9.37 cm, 13.5 cm, and 18.37 cm, respectively.

### 2.5. Data Collection

Full body kinematics were collected using an eight-camera 3D motion capture system recording at 120 Hz (Motion Analysis, Santa Rosa, CA, USA). A Helen Hayes marker set with 30 markers was used; 29 markers were placed on specific bony landmarks to compute the center of mass position [49], while one marker was placed on the belt to determine perturbation onset. Data from reflective markers were low-pass filtered through a fourth order Butterworth filter. The weight exerted on the harness for each trial was measured by the load cell that was synchronized with the motion capture system and connected in series with the harness. Custom written algorithms in MATLAB version 2014b (The MathWorks Inc., Nactick, MA, USA) were used to compute all kinematic variables.

### 2.6. Outcome Measures

**Perturbation outcome**: Perturbation outcome was identified as either backward loss of balance with a fall or a recovery. If the vertical force in the load cell data indicated that more than 30% of participant’s body weight was being supported by the harness at any instant [50], the trial outcome was classified as a fall and further verified by visual inspection of video recordings. [51]. All other trials were classified as recovery. The backward loss of balance outcome was classified when the post-perturbation step (compensatory step) landed posterior to the leading limb [16,52].

**Fall threshold**: The highest intensity reached by the participants in each session wherein participants experienced falls in more than two trials was determined as the fall threshold [30].

**Number of compensatory steps:** The total number of steps taken post-perturbation onset was recorded. The key time instance of recovery step touchdown (TD) was identified when the stepping recovery foot made initial contact with the treadmill belt following the perturbation onset. This was determined by the Z-trajectory of the foot marker upon reaching baseline (similar to the initial positioning during quiet stance), from the heel and metatarsal markers placed over the participant’s foot.

**Multiple step threshold:** The minimum perturbation intensity when participants took more than a single compensatory step was determined as the multiple step threshold [31].

**Postural stability:** Postural stability was determined at the instances of pre-slip touchdown (TD), post-slip lift-off (LO), and post-slip minimum stability (which occurred after liftoff but before the first compensatory step TD) [53,54]. Postural stability was computed as the shortest distance of the instantaneous center of mass (CoM) position and velocity (i.e., CoM state) to the computational threshold boundary for backward balance loss [55,56]. The CoM position was obtained relative to the heel of the posterior-most limb on the ground and was further normalized by the participant’s BoS length (i.e., the foot length during single stance phase and the distance between the posterior-most heel of one limb and the anterior-most toe of the other limb during double stance). The CoM velocity was computed by the first order differentiation of absolute CoM position and was expressed relative to the heel velocity of the posterior-most limb on the ground. It was further normalized by the dimensionless fraction √gh, where *g* is the acceleration due to gravity and *h* is the participant’s body height in meters. Stability values <0 indicate a CoM state below the theoretical boundary for backward loss of balance, signifying a greater likelihood of backward loss of balance, while values between 0 and 1 indicate that the CoM state lies on or within the theoretical boundary for backward loss of balance [53,54].

**Clinical outcome measures:** Scores on clinical measures such as the ABC scale, the Mini-BESTest, BBS, and treadmill natural walking speed were analyzed to determine the effect of treadmill-based gait-slip training. The ABC scale, a 16-item self-reported scale assessing balance confidence during the performance of activities without loss of balance have been known to be valid and reliable among community-dwelling PwCS [57,58,59]. The Mini-BEST test, a 14-item test including reactive balance control and anticipatory adjustments, is a valid and reliable tool to measure dynamic balance in PwCS [60,61]. The BBS scale, a 14-item objective measure is a validated and reliable measure for static balance and fall-risk [62,63].

**Statistical analysis:** To determine the effect of the multisession gait-slip training on fall outcome and the number of compensatory steps, a Friedman’s test was performed including all six gait-slip intensities. Pre- and post-training planned comparison for fall outcome using the paired Chi-square test (McNemar test), and the Wilcoxon’s signed rank test for the number of steps, fall threshold, multiple stepping threshold, and progression of training slip intensity from first to last session were performed. Parametric variables such as pre-slip stability at touchdown, post-slip stability at lift-off, and post-slip minimum stability were analyzed using repeated measures ANOVA, followed by paired *t*-test for planned comparisons. Changes in treadmill natural walking speed and clinical outcomes were analyzed using paired *t*-test. All analyses were performed using SPSS version 24 with a significance level of 0.05. The sample size was estimated based on previous studies on the ActiveStep treadmill-based multi-session perturbation training paradigms, which employed 13 to 15 stroke participants [29].

## 3. Results

### 3.1. Effect of Multisession, Treadmill-Based Gait-Slip Training on Gait-Slips

**Figure 3:**
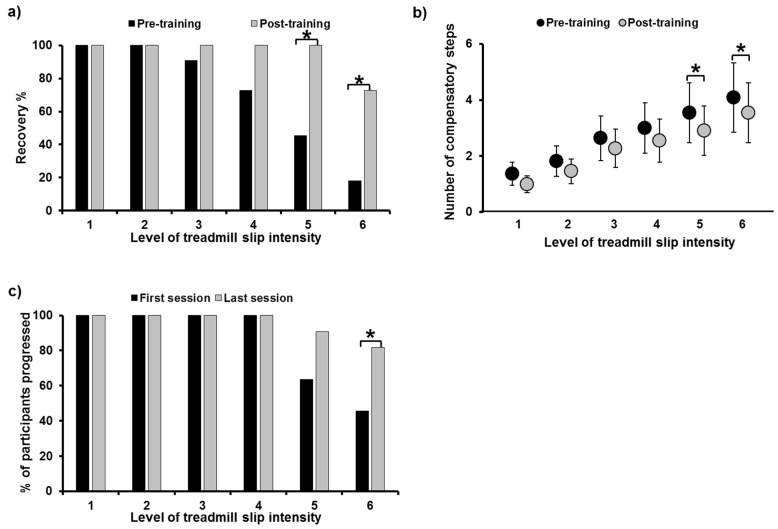
Figure showing (**a**) percentage of recovery and (**b**) number of compensatory steps taken post-perturbation on all six intensities of treadmill-based gait-slips (L1 to L6), comparing pre- and post-training. Figure (**c**) shows the percentage of participants who were able to progress to higher training intensities in the last training session compared to the first training session. *: Significant differences (*p* ≤ 0.05).

Fall outcome: A significant fall reduction from pre- to post-training was noted on all gait-slips (*X*^2^ = 16, *p* = 0.001) (Figure 3a). Since there were no falls on pre- and post-training trials on levels 1, 2, and 3, further analysis was not performed for these trials. There was no significant fall reduction on level 4 (*X*^2^ = 0, *p* = 0.5); however, significant fall reduction was noted on level 5 (*X*^2^ = 4.16, *p* = 0.04) and level 6 (*X*^2^ = 4.16, *p* = 0.04). Fall threshold demonstrated a significant increase in the level of slip intensity when participants experienced a fall at post-training compared to pre-training (*X*^2^ = −2.54, *p* = 0.01).

### 3.2. Number of Compensatory Steps

A significant reduction in the number of compensatory steps from pre- to post-training was noted, including all gait-slips (*X*^2^ = 11.91, *p* = 0.001) (Figure 3b). There was no significant decrease in compensatory steps on level 1 (*Z* = −1.34, *p* = 0.18), level 2 (*Z* = −1.26, *p* = 0.2), level 3 (*Z* = −1.41, *p* = 0.15), and level 4 (*Z* = −0.85, *p* = 0.39), while a near significant decrease was noted on level 5 (*Z* = −1.93, *p* = 0.05) and level 6 (*Z* = −1.89, *p* = 0.05). A step threshold analysis showed a near significant increase on the level of slip intensity when participants performed a multiple stepping response (>1 step) during post-training as compared to pre-training (*X*^2^ = −1.81, *p* = 0.07).

### 3.3. Progression in Training Slip Intensity

In the first and the last training sessions, all participants (*n* = 11) were able to progress to the gait-slip intensity training of level 4 (Figure 3c). There was an increase in the number of participants, from 7 to 10 out of all 11 participants, who were able to progress to level 5 from the first to the last training sessions (*Z* = −1.73, *p* = 0.083), respectively; likewise, a significant increase in participants, from 5 to 9 out of 11 were able to progress to level 6 (*Z* = −2, *p* = 0.046).

**Figure 4:**
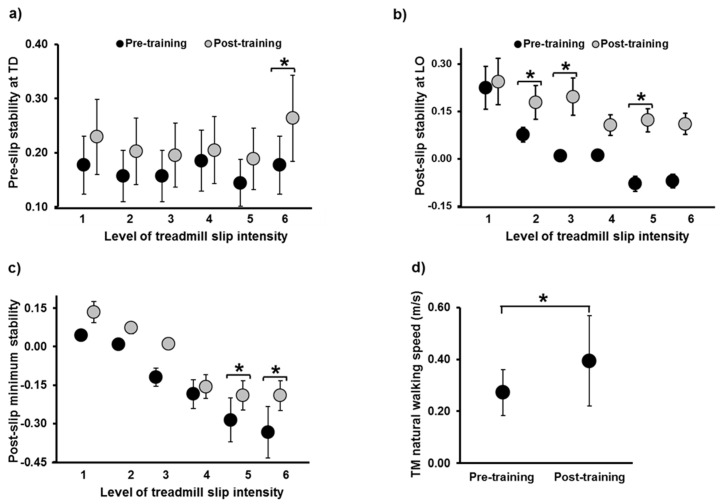
Figure showing (**a**) pre-slip stability at touch down (TD), (**b**) post-slip stability at lift-off (LO), and (**c**) post-slip minimum stability on all six intensities of treadmill-based gait-slips (L1 to L6), comparing pre- and post-training. Stability measures at all instances are dimensionless variables. Figure (**d**) shows the change in treadmill natural walking speed, from pre- to post-training, expressed in meters per second. *: Significant differences (*p* ≤ 0.05). Stability values <0 indicate a CoM state below the theoretical boundary for backward loss of balance, signifying a greater likelihood of backward loss of balance, while values between 0 and 1 indicate that the CoM state lies on or within the theoretical boundary for backward loss of balance.

CoM state stability: Table 2 reports the main effects of trial, intensity, and trial x intensity interaction for repeated measures ANOVA, while Table 3 reports the confidence intervals of planned paired *t*-tests for stability at selected instances. For the pre-slip CoM stability at TD, there was a significant difference in the main effect of trial (*p* ≤ 0.05); however, there was none in the effect of intensity and trial x intensity interaction (*p* > 0.05) (Figure 4a). There was no significant difference in pre-slip stability on levels 1, 2, 3, 4, and 5 (*p* < 0.05); however, a significant increase in stability was noted on level 6 (*p* ≤ 0.05). For the post-slip CoM stability at LO, there was a significant difference in the main effect (*p* ≤ 0.05) and intensity (*p* ≤ 0.05); however, there was none in the trial x intensity interaction (*p* > 0.05) (Figure 4b). There was no significant difference in post-slip stability on levels 1, 4, and 6 (*p* < 0.05); however, a significant increase in stability was noted on levels 2, 3, and 5 (*p* ≤ 0.05). For the post-slip minimum CoM stability, there was a significant difference in the main effect (*p* ≤ 0.05) and intensity (*p* ≤ 0.05); however, there was none in the trial x intensity interaction (*p* > 0.05) (Figure 4c). There was no significant difference in post-slip minimum stability on levels 1, 2, 3, and 4 (*p* < 0.05); however, a significant increase was noted on levels 5 and 6 (*p* ≤ 0.05).

### 3.4. Effect of Multisession, Treadmill-Based Gait-Slip Training on Clinical Measures

There was no significant improvement noted in the ABC, Mini-BESTest, and the BBS scores (*p* > 0.05). However, there was a significant increase in the treadmill walking speed from pre- to post-training (*p* = 0.003, 95% CI = −0.42, −0.11) (Figure 4d).

## 4. Discussion

PwCS demonstrated adaptive improvement in falls and postural stability along with an increase in their treadmill walking speed after the multisession treadmill-based gait-slip training with progressively increasing intensity. However, there was no improvement noted in the clinical outcome measures.

PwCS have demonstrated impaired reactive responses to treadmill-based large-magnitude stance perturbations [16] and further exhibited difficulty to modulate their reactive stepping responses according to the perturbation intensity [64]. While healthy young adults appropriately scaled their responses when exposed to treadmill-induced progressive stance slips, PwCS were unable to modulate their compensatory step length or trunk extension. This resulted in lower post-slip stability at recovery touchdown and eventually increased falls with increasing perturbation intensity. Similarly, at pre-training, PwCS demonstrated no falls on lower intensities of levels 1 and 2; however, PwCS fell on level 3, and falls increased with increasing slip intensity. There was an associated increase in the multiple stepping response and a decrease in post-slip stability (at LO and minimum stability) with increasing slip intensity. These findings are in alignment with previous perturbation studies in PwCS that have indicated the performance of multiple, shorter steps leading to lower postural instability, thereby further contributing to falls [14,15,16]. Thus, exposure to higher perturbation intensities in our paradigm highlighted the deficits in the already impaired reactive balance control of PwCS. It can be postulated that the inability to accurately perceive somatosensory information regarding perturbation intensity or contextual information may have affected the online modification of the triggered motor response, thereby influencing the compensatory stepping response in PwCS [65,66]. These results also support postulations from previous studies regarding the role of higher cortical centers in reactive balance control [67,68,69,70].

With multisession progressive gait-slip training, PwCS demonstrated significant fall reduction and an associated decrease in their multiple stepping response, especially at challenging slip intensities of levels 5 and 6. Conversely, there was an improvement in the post-training fall and the multiple-step threshold. Post-training, PwCS demonstrated a trend of improvement in their proactive gait stability at pre-slip TD, with a significant increase noted at the highest slip intensity of level 6. PwCS also demonstrated reactive improvements in the post-slip stability at LO with a significant increase noted on levels 2, 3, and 5. This result indicates a reduction in the induced loss of balance at comparable intensities post-training. This was further coupled with improved recovery demonstrated through a significant increase in the reactive post-slip minimum stability for the challenging slip intensities of levels 5 and 6. The significant increase in post-slip stability at LO on levels 2 and 3 post-training is indicative of the decreased likelihood of balance loss. This could have further resulted in a reduced reliance on recovery stepping to further improve post-slip minimum stability, which was already more stable with positive values on these levels, thus demonstrating a possible ceiling effect for change. Such rationale could explain the non-significant difference from pre- to post-training on level 2. Additionally, while a trend for change was observed on level 3, the non-significant change on level 4 could be due to this intensity being a transition level, from lower to higher intensity of perturbations; thus, while pre-training values on level 4 were similar to level 3, post-training values were similar or closer to level 5 values. Moreover, with repeated exposure to increasing gait-slip intensity, on the last training session, there was a significant increase in the number of participants progressing to the more challenging intensities of levels 5 (from 45% to 100%) and 6 (from 18% to 73%). Thus, our findings are indicative of the preserved ability of PwCS to undergo adaptive changes after treadmill-based gait-slip training.

The improvement in postural stability and the resulting fall reduction might be associated with a training-induced improvement in reactive stepping, such that PwCS were able to effectively maintain their CoM within the BOS. The treadmill-based perturbations do not allow for any modification to the acceleration-based belt displacement by the participant, as the treadmill is operator-driven and has preprogrammed perturbations unlike the overground perturbation system that allows for a reactive modulation of the slipping kinematics [71,72,73,74]. This results in delivering the predetermined perturbation intensity, thereby predominantly relying on reactive balance control. Training-induced improvement in PwCS indicates the preserved ability of the lesioned central nervous system to effectively utilize motor error to generate appropriate corrective stepping responses through the feedback mechanism. It has been postulated that exposure to repeated perturbations results in the recalibration of the internal representation of stability limits [75,76]. Additionally, the unpredictability associated with the exposure to mixed blocks of varying gait-slip intensities and the progression to higher intensities might help in targeting the training of reactive stepping responses. Our findings substantiated the ability of treadmill-based progressively increasing intensity of gait-slip training to improve the fall-resisting skills of PwCS. Furthermore, we found that the treadmill walking speed significantly increased post-training, which might be associated with decreased apprehension and a locomotor training effect due to the repeated exposure to walking on the treadmill.

Our improvement in multiple-step threshold and gait-speed are consistent with two multisession gait-perturbation training studies performed on stroke [28,30]. Handelzalts [30] found that the sub-acute stroke individuals in the perturbation-based balance training group demonstrated significantly higher multiple-step thresholds compared to those in the weight-shifting and gait-training group. Additionally, Punt [28] found an increase in gait-speed post-perturbation training among PwCS. Thus, our results validate the effectiveness of multisession gait-perturbation training. Given that only four training sessions using higher perturbation intensity were provided in our study, compared to a total of 10 and 12 training sessions provided by Punt [28] and Handelzalts [30] respectively, it is possible that our participant demonstrated significant improvement in laboratory measures, without the need for more training sessions.

While our training resulted in improved kinematic control, there was no significant improvement found in the clinical measures, unlike other multisession studies on stroke [30,31,32]. The failure to show improvement in BBS and mini-BEST test may be due to the task-specific nature of the training that targets reactive balance control during gait, as opposed to these clinical performance-based measures primarily consisting of volitional balance control tasks performed during standing. Secondly, there could have been a ceiling effect, as participants were ambulatory community-dwelling PwCS with already higher BBS scores. Though the Mini-BESTest has a reactive component that employs manual push–pull stance perturbations, it is possible that the manual perturbation intensity might not have been challenging enough for our high functioning participants. While Handelzalts study [30] showed no significant difference in BBS scores, van Duijnhoven et al. [31] demonstrated only a one-point improvement in BBS which was not considered to be clinically relevant. However, it is possible that the trend of improvement in clinical outcomes in these studies might also be attributed to the use of greater training dosage. Moreover, van Duijnhoven et al. [31] demonstrated no immediate pre to post improvement in 6-item ABC scores after five weeks of training. Handelzalts et al. [30], Mansfield et al. [32], and van Duijnhoven et al. [31] provided about 10–12 training sessions (over 2.5–6 weeks) consisting of an average of 60–80 perturbations per session, as compared to our training paradigm consisting of only four training sessions (over four weeks) with 40 perturbations per session. Additionally, our study included community-dwelling, high-functioning PwCS with a low fall-risk indicated by BBS and ABC scores, suggestive of the need for greater dosage and more challenging balance training to induce significant improvements in clinical measures.

Although our training induced positive changes, our findings must be interpreted in light of some limitations. One of the major limitations of our study was the use of a small sample size. Future studies must be performed using larger sample sizes and including a control group to determine the efficacy of our training protocol. Secondly, more training trials at each intensity (greater than four) may be required to induce greater adaptive changes among PwCS. Future studies should be directed toward the examination of the effect of a higher training dosage on reactive balance (falls, postural stability) and clinical measures among such high functioning PwCS. It is possible that incorporating gait-specific clinical outcomes such as Functional Gait Assessment (FGA) and Dynamic Gait Index (DGI) might be more sensitive to demonstrating the changes induced by gait-slip training. Lastly, it is uncertain whether acquired adaptation to such training could be retained for longer-term and translate to the reduction of real-life falls among PwCS. Future studies should determine the effects of such training on real-life falls using fall monitoring, as well as its effects on community ambulation.

Our current findings could be of significant clinical relevance for balance rehabilitation post-stroke, as our custom-designed training based on each participant’s reactive balance capacity demonstrated an improvement in falls and postural stability in PwCS. Such balance training protocol can be performed using a portable and user-friendly motorized treadmill device, which has an enormous potential for easy translation into clinical settings. Lastly, our training protocol was a safe yet challenging means to provide bilateral training to reduce fall-risk.

## 5. Conclusions

The multisession, progressively increasing intensity of treadmill gait-slip training demonstrated promising results with improvement in falls, multiple stepping response, and postural stability, especially at challenging intensities among PwCS. Studies with a larger sample size and control group are needed to validate the clinical efficacy of such training protocols among PwCS. It is also recommended to determine the long-term retention of acquired adaptive changes and generalization effects of untrained tasks to reduce real-life fall-risk.

## Figures and Tables

**Table 1:** Demographics and clinical outcome measures for study participants.

Variable	*n* = 11
Age (years)	63.27 ± 8.2
Height (meters)	1.71 ± 0.08
Weight (kilograms)	83.62 ± 9.54
Gender (Male/Female)	9/2
Chronicity of stroke (years)	6.82 ± 6.49
Impairment level	
CMSA (Leg)	4.91 ± 0.79
CMSA (Foot)	3.64 ± 1.37
AFO/No AFO	7/4
Stroke type (Hemorrhagic/Ischemic)	3/6 *
Balance (BBS)	49.18 ± 3.59
Gait speed (10 m test)(m/s)	0.76 ± 0.24
6 min walk test (meters)	283 ± 83.76

CMSA: Chedoke–McMaster Stroke Assessment scale; AFO: Ankle foot orthosis; BBS: Berg Balance Scale; m/s: meters per second. *: indicates stroke type for 2 participants as missing.

**Table 2:** Repeated measures ANOVA results for pre- and post-slip CoM stability.

	Repeated Measures ANOVA Results		
	Main Effect of Trial	Effect of Intensity	Trial × Intensity Interaction
**Pre-slip CoM stability at Touch down (TD)**	F_(1, 59)_ = 11.84, *p* < 0.001 *	F _(1, 59)_ = 0.55, *p* = 0.73	F_(1, 59)_ = 0.40, *p* = 0.84
**Post-slip CoM stability at Lift-off (LO)**	F_(1, 59)_ = 16.83, *p* < 0.001 *	F_(1, 59)_ = 5.4, *p* < 0.001 *	F_(1, 59)_ = 0.85, *p* = 0.51
**Post-slip minimum stability**	F_(1, 58)_ = 12.15, *p* < 0.001 *	F _(1, 58)_ = 15.23, *p* < 0.001 *	F_(1, 58)_ = 0.42, *p* = 0.83

*: Significant differences (*p* ≤ 0.05) on repeated measures ANOVA results, including all six intensities of gait-slips at pre- and post-training.

**Table 3:** Planned comparison results for pre- and post-slip stability using paired *t*-tests with 95% confidence interval.

	Pre-Training vs. Post-Training Results at Each Intensity		
Intensity	Pre-Slip CoM Stability at TD	Post-Slip CoM Stability at LO	Post-Slip Minimum Stability
**Level 1**	*p* = 0.15, 95% CI = −0.12, 0.02	*p* = 0.78, 95% CI = −0.17, 0.13	*p* = 0.15, 95% CI = −0.24, 0.06
**Level 2**	*p* = 0.06, 95% CI = −0.09, −0.001	*p* = 0.03, 95% CI = −0.19, −0.008 *	*p* = 0.22, 95% CI = −0.17, 0.04
**Level 3**	*p* = 0.42, 95% CI = −0.14, 0.06	*p* = 0.03, 95% CI = −0.36, −0.01 *	*p* = 0.13, 95% CI = −0.31, 0.04
**Level 4**	*p* = 0.43, 95% CI = −0.07, 0.03	*p* = 0.16, 95% CI = −0.23, 0.04	*p* = 0.75, 95% CI = −0.2, 0.15
**Level 5**	*p* = 0.27, 95% CI = −0.14, 0.04	*p* = 0.05, 95% CI = −0.44, 0.01 *	*p* = 0.05, 95% CI = −0.22, 0.004 *
**Level 6**	*p* = 0.03, 95% CI = −0.16, −0.007 *	*p* = 0.12, 95% CI = −0.42, 0.06	*p* = 0.03, 95% CI = −0.27, −0.01 *

*: Significant differences (*p* ≤ 0.05) on planned comparisons using paired *t*-tests to compare differences between each intensity.

## Data Availability

The data presented in this study is available on request from the corresponding author.
